# Anaphylaxis management in a French pediatric emergency department: Lessons from the ANA‐PED study

**DOI:** 10.1002/clt2.12289

**Published:** 2023-08-01

**Authors:** Evangéline Clark, Luciana Kase Tanno, Tram Vo, Brigitte Blanc, Pascal Demoly, Davide Caimmi

**Affiliations:** ^1^ Allergy Unit of the Pneumology Allergy and Thoracic Oncology Service University Hospital of Montpellier Montpellier France; ^2^ IDESP UMR UA11 INSERM University of Montpellier Montpellier France; ^3^ IHU Méditerranée Infection AP‐HM MEPHI Marseille France; ^4^ Pediatric Emergency Department University Hospital of Montpellier Montpellier France

**Keywords:** anaphylaxis, children, emergency department, epinephrine/adrenaline, treatment

## Abstract

**Background:**

Anaphylaxis is a serious systemic hypersensitivity reaction that requires immediate recognition and prompt administration of epinephrine/adrenaline. The present study aimed to assess the appropriateness of epinephrine/adrenaline use in children identified as allergic by physicians in the emergency department (ED) at the time of the reaction, and to identify factors that are possibly associated with epinephrine/adrenaline administration, auto‐injector prescription, and further referral to an allergist.

**Methods:**

We performed a retrospective cross‐sectional study at the pediatric ED of the University Hospital of Montpellier, France. We included all consecutive children who attended the ED between 2016 and 2020 with an allergy‐related diagnosis at discharge.

**Results:**

We included 1056 allergy‐related visits, including 224 (21.2%) with a diagnosis of anaphylaxis at discharge; only 17.0% of them received an epinephrine/adrenaline injection, and 57.1% consulted an allergist after the acute episode. An auto‐injector was prescribed to 63 (28.1%) patients at discharge from the ED. Besides the severity of the clinical presentation, factors associated with a guidelines‐based management of the anaphylactic reaction and with an increased administration rate of epinephrine/adrenaline included presence of asthma symptoms and presence of extended skin reactions.

**Conclusions:**

Our study underlines persistent gaps in the management of pediatric anaphylaxis in ED, focusing on hereby identified levers. By disseminating current knowledge and guidelines on anaphylaxis and allergies, specialists could work together with emergency physicians to establish effective management algorithms and improve anaphylaxis management and care pathways for children experiencing allergic reactions, especially anaphylaxis.

**Trail Registration:**

Clinical Trials, number NCT05112367.

## 1 INTRODUCTION

Anaphylaxis is a serious systemic hypersensitivity reaction, mainly appearing with a rapid onset and being potentially life‐threatening, possibly compromising the respiratory and cardio‐circulatory systems.^1^, ^2^ Its definition has evolved over the past years and has been simplified by multiple publications on this subject^1^, ^2^, ^3^, ^4^ to avoid capturing severe cases only. In 2020, the World Allergy Organization (WAO) defined anaphylaxis as an allergic hypersensitivity reaction involving at least two organs, without the need for patients to present signs of hypotension and/or shock.^4^ European data show a lifetime prevalence rate of anaphylaxis of around 0.3% (95% confidence interval (CI) 0.1–0.5), while incidence rates for all causes of anaphylaxis range from 1.5 to 7.9 per 100,000 person/year.^5^ In children, the incidence of anaphylaxis ranges from 1 to 761 cases per 100,000 person/year.^6^ Fatalities due to pediatric anaphylaxis remain relatively low over the last decades in France: between 1979 and 2014, the anaphylaxis mortality rate was 0.077 per million children per year, with a decrease of 7.8% between 1979 and 1985 and 2001–2005.^7^

Allergic reactions, including anaphylaxis, are likely to be more frequent in the community. Diagnosis may be challenging, for example, in the absence of an obvious trigger or when symptoms are atypical or rapidly resolving, resulting in an underuse of epinephrine/adrenaline.^8^, ^9^

Early recognition of the reaction and prompt administration of epinephrine/adrenaline remain the cornerstone of the management of anaphylaxis.^1^, ^2^, ^3^, ^4^, ^7^, ^8^, ^9^, ^10^ It is now well known that a delayed epinephrine/adrenaline administration (or no administration at all) is associated with increased mortality and a risk of biphasic reactions.^10^ Also, an epinephrine/adrenaline auto‐injector (EAI), in those countries in which such an option is available, should be prescribed before discharge from the emergency department (ED) to any patient at risk of presenting a further episode of anaphylaxis; also, patients and their caregivers should receive personalized education on its use.^2^, ^3^

Despite the existence of national and international guidelines and awareness campaigns on anaphylaxis, epinephrine/adrenaline continues to be underused,^11^, ^12^ and antihistamines and corticosteroids are still prescribed as a first line treatment.^13^ Several barriers for the appropriate use of epinephrine/adrenaline by physicians in the management of anaphylaxis are suggested, such as a lack of knowledge about epinephrine/adrenaline administration, fear of possible drug‐related side effects, misdiagnosis and/or under‐diagnosis or, less frequently, over‐diagnosis.^12^, ^14^ Misdiagnosis may be explained by benign clinical presentation, transient symptoms, or unusual symptoms (such as absence of skin symptoms), and difficulty in communication, if the child cannot describe the symptoms.

The present study aimed to assess the appropriateness of epinephrine/adrenaline use in children identified as allergic by physicians in the ED at the time of the reaction, and to identify factors possibly associated with ED epinephrine/adrenaline administration, EAI prescription at discharge, and further referral to an allergist.

## 2 METHODS

### 2.1 Study design, setting, and population

We conducted a cross‐sectional and observational study at the pediatric ED of the University Hospital of Montpellier (France). We included all consecutive children who attended the ED between 2016 and 2020, with an allergy‐related discharge diagnosis, based on the tenth revision of the International Classification of Diseases (ICD‐10) (Electronic Repository Table A). Patients were excluded if medical records were missing or unavailable.

The study was approved by the Institutional Review Board of Montpellier (IRB‐MTP_2021_10_202100955) and registered on ClinicalTrials.gov (NCT05112367).

### 2.2 Collected data

Besides diagnosis, patients' data were manually extracted through record review by two independent allergists: demographic data, clinical manifestations, previous clinical history with allergic disorder, allergy therapy prescribed during ED access (including possible treatment provided by paramedics/physicians in the ambulance), post‐emergency discharge prescriptions and therapeutic education. As for clinical manifestations, we defined the presence of hypotension as a decrease of 30% of patients' systolic blood pressure compared to the basal value assessed either at their arrival at the ER or on the ambulance before admission. Based on available data, two independent allergists confirmed or not the diagnosis of symptoms possibly related to an allergic reaction and its severity (i.e., anaphylactic, or not) according to the Ring and Messmer classification,^15^ adapted^16^ and accepted by the WHO, in the ICD‐11 classification^17^ (Electronic Repository Table B).

Possible disagreement between the two allergists was resolved through discussion with a third specialist to reach a final consensus. We then differentiated patients presenting with grade I reaction from those with anaphylaxis; we defined as suffering from anaphylaxis only patients presenting with grade II and above symptoms.

All families were contacted by phone to verify if they were referred/consulted an allergist after the acute episode.

### 2.3 Outcomes of the study

The primary outcome was the appropriateness of epinephrine/adrenaline injection to children identified as allergic by emergency physicians in the pediatric ED of Montpellier.

The appropriateness assessment was based on the European Academy of Allergy and Clinical Immunology (EAACI) guidelines and the currently accepted international definition of anaphylaxis^2^, ^17^, ^18^, ^19^:

- all patients experiencing anaphylaxis should be treated with epinephrine/adrenaline;
- all patients at risk of anaphylaxis are recommended to receive a prescription of EAI;
- epinephrine/adrenaline should not be administered to children who do not experience anaphylaxis.

Secondary objectives were to identify factors associated with ED epinephrine/adrenaline administration, EAI prescription, and referral to an allergist in children experiencing anaphylaxis.

### 2.4 Statistical analysis

Qualitative variables were evaluated as frequencies and percentages, and comparisons of the qualitative data between treated and untreated patients with epinephrine/adrenaline were carried out using chi‐square or Fisher's exact test for small samples. Quantitative variables were evaluated as median and interquartile ranges and assessed with the Wilcoxon rank‐sum test since they were not normally distributed. Data were considered statistically significant if *p‐value* was ≤0.05. Univariate and multivariate logistic regression models were used to assess clinical factors associated with ED epinephrine/adrenaline administration, EAI prescription, and referral to an allergist in children experiencing anaphylaxis; odds ratios (ORs) were expressed with 95% CIs. Variables associated with an outcome in the univariate analysis (*p‐value* <0.25) were considered for the multivariate model, and the final model was selected using stepwise regression (*p‐value* <0.05) with Akaike Information Criterion (AIC); discrimination was measured by the area under the curves (AUC). Correlations between independent variables were also assessed before the selection of the final model. In the multivariable model, both outcome and predictor variables were dichotomized into ‘Yes’ or ‘No’ responses, along with the variable “sex” that was dichotomized into ‘Female’ or ‘Male’. All analyses were performed using SAS version 9.4 (SAS Institute Inc, Cary, NC, USA).

## 3 RESULTS

### 3.1 Baseline demographics

**FIGURE 1:**
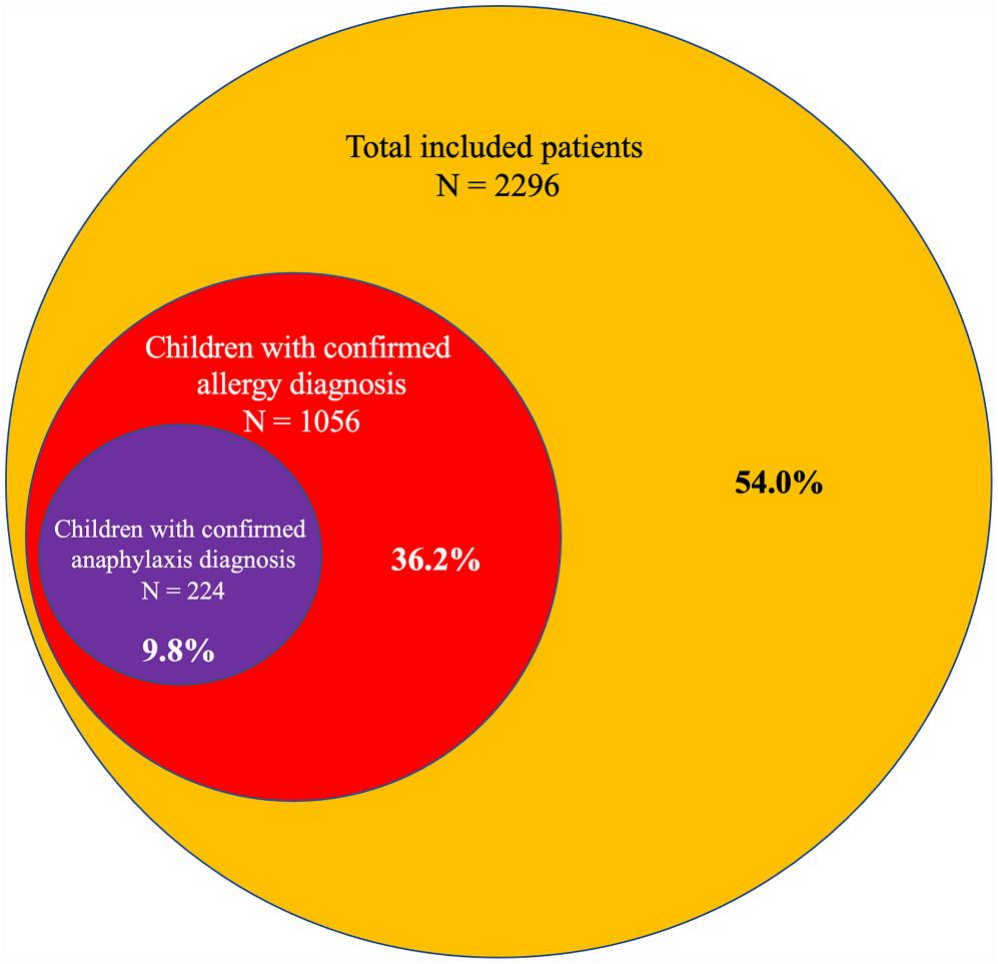
Patients included in our study, Venn diagram. The total number of patients identifies those with the International Classification of Diseases (ICD‐10) diagnosis possibly evocative of an allergic reaction. The two subgroups are composed of children experiencing an allergic reaction and an anaphylactic one, based on an examination of data from their medical charts.

Of the 2296 children included and diagnosed using the ICD‐10 codes as presenting with an allergic reaction by ED physicians from 2016 to 2020, we confirmed that symptoms were evocative of an allergic reaction only in 1056 patients (46.0%), including 832 patients (78.8%) presenting with grade I hypersensitivity reactions and 224 (21.2%) with anaphylaxis. Concerning these 224 patients, all families answered our phone calls, mainly investigating EAI prescription and allergist referral upon discharge. The Venn diagram shown in Figure 1 describes the study population.

The median age was 53 months for the total population (interquartile range, 20–110 months), and 89 months for children presenting with anaphylaxis (interquartile range, 51–159 months). More than a half of them (53.0% and 57.1%, respectively) were males. 40.2% of children suffering from anaphylaxis reported a history of food allergy. 27.7% consulted during the weekends and 47.3% at night. Other baseline demographic characteristics are listed in Table 1.

**TABLE 1:** Characteristics of the included patients.

	Anaphylaxis group		*p*‐value^a^	Non‐anaphylaxis group		*p*‐value^b^
	*N* = 224			*N* = 2072		
	*T	**NT		*T	**NT	
	*N* = 38	*N* = 186		*N* = 5	*N* = 2067	
Sex, *N* (%)			*0.4109*			*0.3775*
Female	14 (36.8)	82 (44.1)		1 (20.0)	983 (47.6)	
Male	24 (63.2)	104 (55.9)		4 (80.0)	1084 (53.4)	
Age (months), Med [Q1‐Q3]	106 [51–153]	89 [51–159]	*0.8111*	94 [8–187]	48 [19–103]	*0.2398*
ICD‐10 codes, *N* (%)						
Anaphylactic shock. Unspecified	14 (36.9)	12 (6.5)		0	2 (0.1)	
Allergic reaction, unspecified	6 (15.8)	69 (37.1)		3 (60.0)	366 (17.7)	
Urticaria	3 (7.9)	22 (11.8)		1(20.0)	1226 (59.3)	
Anaphylactic shock or reaction due to unspecified food	1 (2.6)			0	0	
Quincke's edema	12 (31.6)	22 (11.8)		1 (20.0)	28 (1.4)	
Upper respiratory tract hypersensitivity reaction	2 (5.3)	6 (3.2)		0	3 (0.2)	
Localized edema	0	3 (1.6)		0	243 (11.8)	
Generalized edema	0	0		0	3 (0.2)	
Edema, unspecified	0	0		0	13 (0.6)	
Edema of larynx	0	1 (0.5)		0	0	
Allergic rhinitis	0	0		0	183 (8.9)	
Previous clinical history with allergic disorder, *N* (%)						
Unspecified allergy	0	4 (2.2)	*1.00*	1 (20.0)	16 (0.8)	***0.0404***
Respiratory allergy	10 (26.3)	35 (18.8)	*0.2931*	0	138 (6.7)	*1.00*
Food allergy	22 (57.9)	68 (36.6)	***0.0145***	3 (60.0)	119 (5.8)	***0.0018***
Drug allergy	1 (2.6)	7 (3.8)	*1.00*	0	22 (1.1)	*1.00*
Medical follow‐up, *N* (%)						
Already followed by an allergist	21 (55.3)	73 (39.3)	*0.0683*	3 (60.0)	137 (6.6)	***0.0027***
Already followed for the same allergy	13 (34.2)	48 (25.8)	*0.2889*	2 (40.0)	69 (3.3)	***0.0108***
No medical follow‐up, *N* (%)	17 (44.7)	113 (60.8)		2 (40.0)	1930 (93.4)	
Time and date of arrival at the emergency room, *N* (%)						
Weekend (Saturday‐Sunday)	9 (23.7)	53 (28.5)	*0.5459*	2 (40.0)	731 (35.4)	*1.00*
Week (Monday‐Friday)	29 (76.3)	133 (71.5)		3 (60.0)	1336 (64.6)	
Night (8pm‐7.59am	13 (34.2)	93 (50.0)	*0.0757*	3 (60.0)	1020 (49.4)	*0.6834*
Day (8am‐7.59 pm)	25 (65.8)	93 (50.0)		2 (40.0)	1047 (50.7)	

*Note*: Bold numbers are those statistically significant.

^a^ Comparison between anaphylaxis‐treated by epinephrine/adrenaline group and anaphylaxis‐not treated by epinephrine/adrenaline group was performed using the chi2 test or Fisher's exact test and Wilcoxon test.

^b^ Comparison between non‐anaphylaxis‐treated by epinephrine/adrenaline group and non‐anaphylaxis‐non treated by epinephrine/adrenaline group was performed using the chi2 test or Fisher's exact test and Wilcoxon test.

*T: treated by epinephrine/adrenaline.

**NT: not treated by epinephrine/adrenaline.

### 3.2 Clinical presentation

The clinical symptoms of the 2296 patients and of the subgroup of 224 children suffering from anaphylaxis are presented in Table 2.

**TABLE 2:** Clinical manifestations according to epinephrine/adrenaline administration.

	Anaphylaxis group		*p*‐value^a^	No anaphylaxis group		*p*‐value^b^
	*N* = 224			*N* = 2072		
	*T	**NT		*T	**NT	
	*N* = 38	*N* = 186		*N* = 5	*N* = 2067	
Skin, subcutaneous tissue and mucosa, *N* (%)						
Localized urticaria	1 (2.6)	6 (3.2)	*1.00*	1 (20.0)	139 (6.7)	*0.2954*
Generalized urticaria	30 (79.0)	138 (74.2)	*0.5374*	0	1075 (52.0)	***0.0257***
Localized angioedema	16 (42.1)	72 (38.7)	*0.6961*	2 (40.0)	437 (21.1)	*0.2871*
Generalized angioedema	11 (29.0)	17 (9.1)	***0.0021***	2 (40.0)	67 (3.2)	***0.0102***
Respiratory tract, *N* (%)						
Sensation of dyspnea	9 (23.7)	82 (44.1)	***0.0196***	0	17 (0.8)	*1.00*
Wheezing/bronchospasm	24 (63.2)	46 (24.7)	***<0.0001***	1 (20.0)	7 (0.3)	***0.0192***
Rhino‐conjunctivitis	4 (10.5)	34 (18.3)	. *2459*	0	74 (3.6)	*1.00*
Gastrointestinal tract, *N* (%)						
Vomiting or nausea	13 (34.2)	69 (37.1)	*. 0.7365*	0	27 (1.3)	*1.00*
Diarrhea	0	25 (13.4)	*0.2268*	0	13 (0.6)	*1.00*
Cardiovascular system, *N* (%)						
Hypotension	12 (31.6)	27 (14.5)	***0.0115***	0	0	
Central nervous system, *N* (%)						
Feeling of uneasiness	9 (23.7)	25 (13.4)	*0.1088*	0	6 (0.3)	*1.00*
Convulsions	1 (2.6)	3 (1.6)	*0.5272*	0	0	
Loss of consciousness	1 (2.6)	0	*0.1696*	0	12 (0.6)	*1.00*
Severity***, *N* (%)			***0.0115***			
Grade I	‐	‐		5 (100.0)	779 (37.7)	
Grade II	26 (68.4)	159 (85.5)		‐	‐	
Grade III	12 (31.6)	27 (14.5)		‐	‐	
Grade IV	0	0		‐	‐	
No allergy, *N* (%)	‐	‐		‐	1240 (60.0)	
Non‐IgE mediated allergic disorders, *N* (%)^2^	‐	‐		‐	48 (2.3)	

*Note*: Bold numbers are those statistically significant.

^a^ Comparison between anaphylaxis‐treated by epinephrine/adrenaline group and anaphylaxis‐not treated by epinephrine/adrenaline group was performed using the chi2 test or Fisher's exact test and Wilcoxon test.

^b^ Comparison between non‐anaphylaxis‐treated by epinephrine/adrenaline group and non‐anaphylaxis‐non treated by epinephrine/adrenaline group was performed using the chi2 test or Fisher's exact test and Wilcoxon test.

*T: treated by epinephrine/adrenaline.

**NT: not treated by epinephrine/adrenaline.

***Severity assessed according to Ring and Messmer's classification.

At the ED admission, 185 patients (82.6%) presented with grade II anaphylaxis, and 39 (17.4%) with grade III anaphylaxis. No death from anaphylactic reactions was observed in the included population. The main presenting symptom, in patients with anaphylaxis, was generalized urticaria, recorded in 168 patients (75.0%).

Considering WAO criteria,^4^ 97% of our patients presenting with anaphylaxis fulfilled criterion 1 (51% for criterion 1a, 4% for criterion 1b, and 20% for criterion 1c; 5% fulfilled both criteria 1a and 1b, 12% fulfilled criteria 1a and 1c, 2% fulfilled criteria 1b and 1c, and 3% fulfilled criteria 1a, 1b, and 1c); 3% could be classified as presenting with criterion 2.

### 3.3 Appropriateness of reaction management and discharge

At the emergency room, most children with anaphylaxis were treated with oral antihistamines (72.8%) and oral corticosteroids (61.6%); intramuscular epinephrine/adrenaline was used in 17.0% of them. Epinephrine/adrenaline was more frequently prescribed in patients experiencing anaphylaxis with generalized angioedema (39.3% vs. 13.8%, *p‐value* 0.021), or with asthma (34.3% vs. 9.1%, *p‐value* <0.0001). Epinephrine/adrenaline administration was higher for grade III anaphylaxis than for grade II anaphylaxis, even though the difference between groups was not statistically significant (30.8% vs. 14.1%, *p‐value* 0.115). On the other hand, 5 of 2072 (0.24%) without anaphylaxis received an injection of epinephrine/adrenaline.

Among children with anaphylaxis, epinephrine/adrenaline was more often used in children with a reported history of food allergy (*p‐value* 0.145), or food anaphylaxis (*p‐value* 0.018) or being followed by an allergist (2.1% vs. 0.1%, *p‐value* 0.027).

**FIGURE 2:**
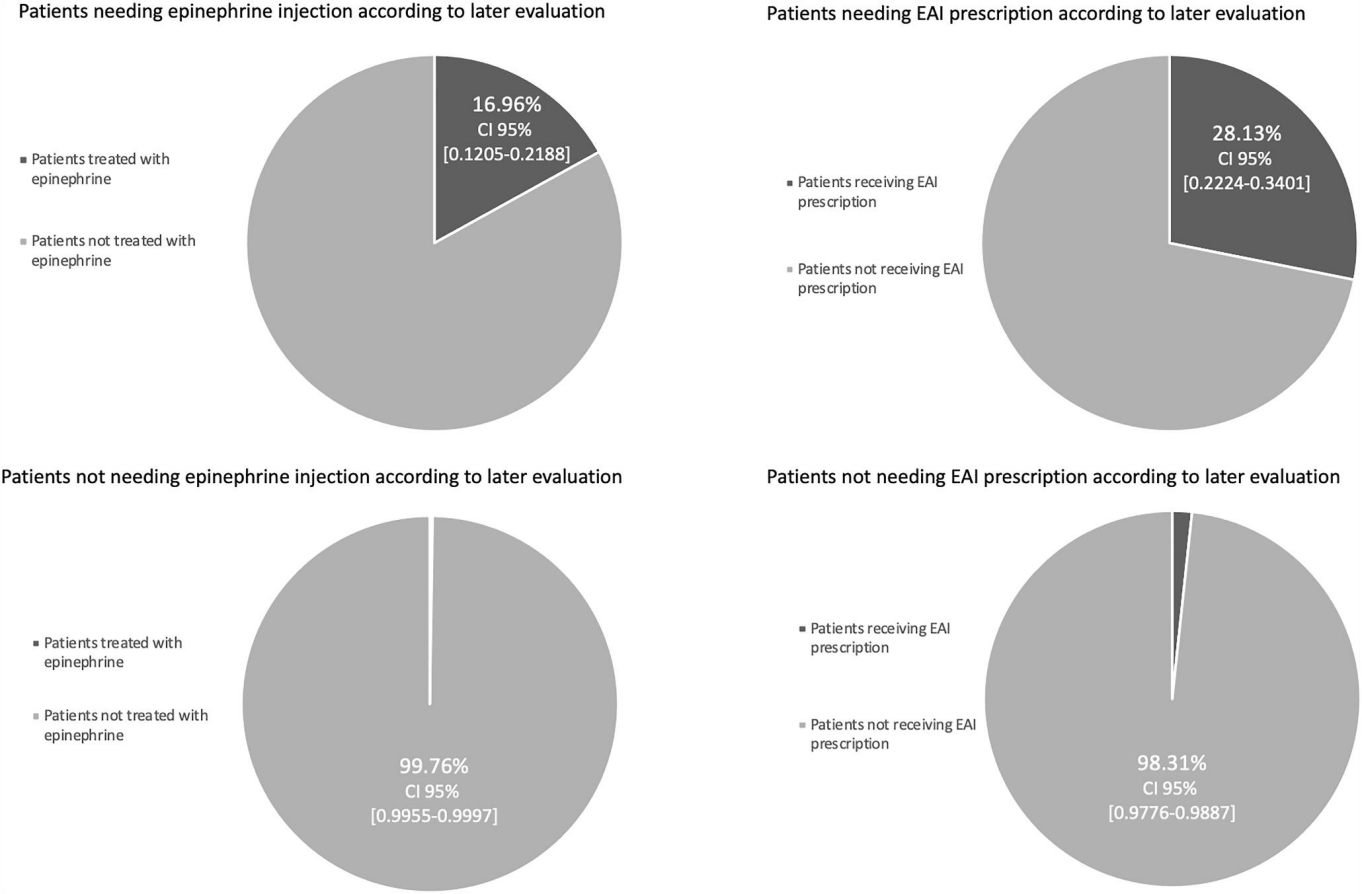
Appropriateness of epinephrine/adrenaline administration to children with allergist–confirmed anaphylaxis in the pediatric emergency department (ED) of Montpellier, and of its prescription upon discharge from the ED. EAI: epinephrine/adrenaline auto‐injector; CI: confidence interval (CI).

Overall, 28.1% of children with anaphylaxis were discharged with a prescription for an EAI and 50.0% of them received therapeutical education. 57.1% children with anaphylaxis were referred to an allergist after the acute episode. In 35 children not experiencing anaphylaxis (1.7%), an EAI was prescribed at discharge. Figure 2 summarized main outcome results, and Table 3 shows treatment management at ED and at discharge.

**TABLE 3:** Treatment administration during emergency and prescription at discharge according to epinephrine/adrenaline administration.

	Anaphylaxis group		*p*‐value^a^	No anaphylaxis group		*p*‐value^b^
	*N* = 224			*N* = 2072		
	*T	**NT		*T	**NT	
	*N* = 38	*N* = 186		*N* = 5	*N* = 2067	
Treatments received in ED						
Glucocorticoids IV or IM	19 (50.0)	18 (9.7)	***<0.0001***	0	12 (0.6)	*1.00†*
Glucocorticoids oral	19 (50.0)	119 (64.0)	*0.1064*	4 (80.0)	536 (25.9)	***0.0181***
Antihistamine IV	9 (23.7)	15 (8.1)	***0.0089***	0	21 (1.0)	*1.00*
Antihistamine oral	22 (57.9)	141 (75.8)	***0.0238***	4 (80.0)	1185 (57.3)	*0.4014†*
Epinephrine/adrenaline Inhalation	5 (13.2)	27 (14.5)	*0.8274*	0	21 (1.0)	*1.00*
Oxygen	11 (29.0)	5 (2.7)	***<0.0001***	0	1 (0.1)	*1.00*
Salbutamol	13 (34.2)	31 (17.0)	***0.0131***	1 (20.0)	15 (0.7)	***0.0381***
Discharge care post‐emergency (emergency kit)						
Antihistamines	16 (42.1)	44 (23.7)	***0.0193***	1 (20.0)	137 (6.6)	*0.2918*
Glucocorticoids	16 (42.1)	40 (21.5)	***0.0075***	1 (20.0)	49 (2.4)	*0.1151*
Salbutamol	1 (2.6)	2 (1.1)	*0.4291*	0	1 (0.1)	*1.00*
Epinephrine/adrenaline	15 (39.5)	48 (25.8)	*0.0877*	2 (40.0)	33 (1.6)	***0.0027***
Discharge care post‐emergency (medical advice)	20 (52.6)	92 (49.5)	*0.7218*	2 (40.0)	324 (15.7)	*0.1783*
Referral to an allergy specialist			*0.1138*			***0.0089***
University hospital	14 (36.8)	61 (32.8)		0	147 (7.1)	
Allergist in town	14 (36.8)	39 (21.0)		2 (40.0)	148 (7.2)	

*Note*: Bold numbers are those statistically significant.

^a^ Comparison between anaphylaxis‐treated by epinephrine/adrenaline group and anaphylaxis‐not treated by epinephrine/adrenaline group was performed using the chi2 test or Fisher's exact test and Wilcoxon test.

^b^ Comparison between non‐anaphylaxis‐treated by epinephrine/adrenaline group and non‐anaphylaxis‐non treated by epinephrine/adrenaline group was performed using the chi2 test or Fisher's exact test and Wilcoxon test.

*T: treated by epinephrine/adrenaline.

**NT: not treated by epinephrine/adrenaline.

Factors associated with epinephrine/adrenaline administration, EAI prescription, and referral to an allergist in children with anaphylaxis.

A multivariate logistic regression model was fitted to determine factors that were associated with ED epinephrine/adrenaline administration (Table 4), EAI prescription (Electronic Repository Table C), and referral to an allergist (Electronic Repository Table D) in children with anaphylaxis.

**TABLE 4:** Univariate and multivariate logistic regression analysis by stepwise selection of factors associated with epinephrine/adrenaline administration in children with anaphylaxis.

	Univariate analysis			Multivariate analysis		
	OR	95% CI	*p*‐value	OR	95% CI	*p*‐value
Sex (F vs. M)	0.740	0.36–1.52	0.4120			
Age (months)	1.001	1.00–1.01	0.8057			
Previous clinical history with allergic disorder						
Respiratory allergy	1.541	0.69–3.47	0.2957			
Food allergy	2.386	1.17–4.85	**0.0163**	3.013	1.33–6.84	**0.0084**
Drug allergy	0.692	0.08–5.79	0.7337			
No previous clinical history	0.399	0.19–0.85	0.0174			
Medical follow‐up						
Already followed for an allergy	1.912	0.95–3.87	**0.0711**			
Time and date of arrival at the emergency room						
Weekend (Saturday‐Sunday)	0.779	0.35–1.76	0.5467			
Night (8pm‐7.59am)	0.520	0.25–1.08	**0.0788**			
Skin, subcutaneous tissue and mucosa						
Localized urticaria	1.152	0.57–2.34	0.6963			
Generalized urticaria	1.304	0.56–3.04	0.5384			
Localized angioedema	1.152	0.57–2.34	0.6963			
Generalized angioedema	4.050	1.71–9.57	**0.0014**	6.167	2.23–17.09	**0.0005**
Respiratory tract						
Sensation of dyspnea	0.394	0.18–0.88	**0.0277**			
Wheezing/bronchospasm	5.217	2.49–10.92	**<0.0001**	7.614	3.22–18.01	**<0.0001**
Rhino‐conjunctivis	0.526	0.18–1.58	0.2526			
Gastrointestinal tract						
Vomiting or nausea	0.882	0.42–1.84	0.7366			
Central nervous system						
Feeling of uneasiness	1.999	0.85–4.72	**0.1139**			
Convulsions	1.649	0.17–16.29	0.6688			
Severity						
Grade III versus .II	2.718	1.23–6.03	**0.0139**	4.728	1.73–12.91	**0.0024**

*Note*: Bold numbers are those statistically significant.

In the final model, a reported history of food allergy, symptoms of generalized angioedema, asthma, and grade III anaphylaxis were associated with the injection of epinephrine/adrenaline with odds‐ratio (OR) values of 3.013, 6.167, 7.614, and 4.728, respectively. All *p‐values* were <0.05. For the ROC curve analysis, the final model produced a good AUC of 81.2% with the smallest Akaike Information Criteria (AIC).

As for EAI prescription, the stepwise model retained four variables in the final model, with an acceptable AUC of 73.0%. These variables were the presence of urticaria, asthma exacerbation, treatment with oral corticosteroids, and therapeutic education provided before discharge.

The multivariable analysis final model showed that patients receiving therapeutic education before ED discharge were more likely to be referred to an allergist (OR, 4.175; 95% CI, 2.284–7.632); also, concerning patients' sex, girls tended to be less frequently referred to an allergist than boys (OR, 0.522; 95% CI, 0.290–0.942). For the ROC curve analysis, the final model produced an acceptable AUC of 80.3% rate.

## 4 DISCUSSION

With the rise of allergy incidence and prevalence, especially in Western countries and in children,^6^ physicians should be aware of how to appropriately manage patients presenting with anaphylactic symptoms, especially in emergency settings. We present the results of a retrospective cross‐sectional study with a large sample size that explored the *real‐life* management of anaphylaxis in children admitted to the pediatric ED of the University Hospital of Montpellier. Every year, 31000 children visit this department. To our knowledge, this is the first cross‐sectional study to explore the management of anaphylaxis in children in a French ED, including a control group of patients experiencing a Grade I allergic reaction, and a follow‐up interview to clarify and complete data. Other studies were recently performed in the US^20^, ^21^ and Australia.^22^ A prospective study performed in Denmark included patients assessed at the ER and then evaluated for a diagnostic confirmation at an allergy unit, and aimed at characterizing these patients,^23^ focusing also on the reaction management at the ER.^24^

Among the 2296 children evaluated, only 1056 were confirmed as presenting with a (likely) allergic reaction. To evaluate the possible allergic origin of the presented symptoms, each chart was evaluated by two independent allergists, and by a third one in case of disagreement. The fact that 1240 children were excluded from the analysis underlines once again that allergic and hypersensitivity diseases are poorly and not specifically represented in the ICD‐10 classification, which was the origin of the need to update this aspect in the ICD‐11 version^25^ Indeed, diagnosis such as “urticaria (L50‐L54)” was included in the original database. Nevertheless, such an unspecific diagnosis in children is often due to non‐allergic conditions. Therefore, these children were not correctly classified due not necessarily to a possible misdiagnosis in the ER department, but mainly to the difficulty in classifying them, according to the ICD‐10.

As it has been previously shown,^19^, ^26^ we recorded an underuse of epinephrine/adrenaline at the time of the anaphylactic reaction (16.96%). Nevertheless, our results show a lower rate compared with those from the European anaphylaxis register (27.1%).^8^ A study conducted in Marseille (France) in 2019 used Sampson's criteria to select patients possibly presenting with anaphylaxis, but then classified them following Ring and Messmer's scale, as we did for severity and treatment management. The Authors reported that 84.8% of patients presenting with Grade III anaphylaxis received epinephrine/adrenaline,^14^ which is a much higher rate compared to our population (30.8%). Such a result highlights the fact that discrepancies exist not only between countries but even within the same country.

Innovation has been brought to our study by identifying factors associated with epinephrine/adrenaline administration, besides severity.^14^ Indeed, we showed that clinicians are more prone to administer epinephrine/adrenaline in patients with respiratory symptoms (asthma), generalized angioedema, previous history of food allergy, and grade III anaphylaxis. In our population, age is not associated with under‐ or over‐use, as previously suggested in other studies.^27^ Probably, the presence of lower respiratory symptoms and/or of generalized edema makes emergency physicians more likely to administer epinephrine/adrenaline in acute settings since they might associate these symptoms with a possible evolution toward fatality. We could also suggest that food allergy, known to be associated with a higher risk of anaphylaxis, could be seen by ED doctors as a risk factor for severe forms and make them more prone to promptly administer the appropriate treatment. Our data cannot explain why in 83.0% of children presenting with anaphylaxis, epinephrine/adrenaline was not administered. This group also unexpectedly included 27 children with anaphylaxis and hypotension. We could simply speculate that anaphylactic symptoms may be interpreted as due to a spectrum of possible differential diagnosis, typical of the pediatric population and that, once again, either anaphylaxis is not promptly diagnosed by non‐specialists or that education of allergy symptoms and treatments is still lacking among non‐allergist physicians.

Also, as for the acute treatment administration, more than half of the patients with anaphylaxis in our study received corticosteroids and/or antihistamines, which emphasizes that these drugs are still prescribed as first‐line treatments in this setting. The overuse of antihistamines and corticosteroids for the acute treatment of anaphylaxis has been found in other studies.^14^, ^27^, ^28^, ^29^, ^30^, ^31^ However, these drugs are not recommended and not effective in anaphylaxis management^2^, ^3^, ^13^, ^32^, ^33^, ^34^ and may delay the administration of epinephrine/adrenaline.

Following current guidelines, EAI should be prescribed to all children with a history of anaphylaxis.^2^, ^3^ Yet, in our population, only a minority of them (28.1%) were discharged from the ED with such a prescription. In contrast to acute epinephrine/adrenaline administration, the severity of the reaction was not associated with an increase in discharge prescriptions. Several studies have shown higher rates of EAI prescription ranging from 30% to over 90%.^14^, ^35^, ^36^ Also, differences have been highlighted when considering a referral to a specialist for a follow‐up visit: while this was the case for 57.1% of our patients, data in the literature show percentages ranging from 31.3% to 44.2%.^14^, ^37^ Our higher rate can be biased by the fact that we contacted all families systematically by phone to verify such information.

One last aspect that should be pointed out by our results is that even though we state that epinephrine/adrenaline was underused, there were no fatalities in patients presenting with anaphylaxis, and all children were discharged, either at the ER or after hospitalization, with symptoms resolved. There are no solid data on the treatment of anaphylaxis with adrenaline versus placebo, but such a study would not be possible to perform. Further, the severity of a future anaphylactic reaction is difficult to foresee.^38^, ^39^ Also, there is insufficient evidence to conclude that adrenaline is lifesaving, especially considering that the baseline risk of anaphylaxis fatality is exceptionally low at baseline if patients receive any treatment.^40^ Indeed, one could argue that any hypersensitivity reaction might be self‐resolutive (owing to the person own secretion of endogenous natural adrenaline) or be reduced in severity by antihistamines alone, but this hypothesis is merely speculative. From a risk management point of view, considering the mechanisms of action of the drug and of the immunological reaction, it is still to be considered as a safer and the best recommended option to promptly treat any anaphylactic reaction with adrenaline.

Our study presents some limitations. First, being a retrospective cross‐sectional study, certain clinical information could be missing from the medical record, even after contacting the families by phone. Moreover, our work is based on data from a single center; the generalizability of the results should be examined via additional multicenter and/or longitudinal studies. Also, we retrospectively classified hypersensitivity allergic reactions following Ring and Messmer's classification and many other different classifications have been proposed after the cited one. Even though this classification was initially applied to drugs hypersensitivity reactions, it has been widely used since its original publication to classify severity reactions from any type of allergen. In our cohort, we did not focus on the type of allergen eliciting the symptoms. Therefore, we preferred to use Messmer's method since it is widely accepted, even though it could score for a milder severity score in food allergy compared to Sampson's classification.^41^ Moreover, this classification aligns with the current definition of anaphylaxis proposed by the WHO classification ICD‐11.^16^ Finally, a high rate of non‐allergic reactions (54.0%) was also reported in our study, underlining the limitations of the use of administrative codes (ICD‐10), which might lead to misclassification and misdiagnosis.^42^ The currently deployed version of ICD (ICD‐11) now includes “Allergic and hypersensitivity conditions” section (in the “Immune System Diseases” chapter)^16^, ^17^, which will allow a more accurate diagnosis and follow‐up of anaphylaxis.

In conclusion, the present study helps better understand how anaphylaxis is managed at the emergency room in children based on *real‐life* data and fills the gaps in current knowledge. We demonstrated the need to strengthen local and/or regional training on anaphylaxis management and dissemination of current guidelines to emergency physicians to promptly administer the adapted therapy, and avoid unneeded drugs. Medical campaigns and training events could help better handle anaphylaxis and help doctors rapidly identify it, regardless of the wide variety of possible symptoms. Also, clinicians should be more at ease in referring children at high risk of anaphylaxis to a specialist for a follow‐up visit after the acute reaction. A closer relationship between emergency departments and allergy departments could therefore improve the management of allergies and provide a real and effective support to children and families dealing with these conditions.

## AUTHOR CONTRIBUTIONS

**Evangeline Clark**: Data curation (equal); formal analysis (lead); investigation (equal); project administration (equal); resources (equal); writing – original draft (lead); writing – review & editing (equal). **Luciana Kase Tanno**: Conceptualization (equal); data curation (equal); supervision (equal); validation (equal); writing – review & editing (equal). **Tram Vo**: Formal analysis (equal); investigation (equal); project administration (equal); resources (equal). **Brigitte Blanc**: Data curation (equal); investigation (equal); project administration (equal). **Pascal Demoly**: Methodology (equal); supervision (equal); validation (equal); writing – review & editing (equal). **Davide Caimmi**: Conceptualization (equal); data curation (equal); investigation (equal); project administration (equal); supervision (lead); validation (equal); writing – original draft (equal); writing – review & editing (lead).

## CONFLICT OF INTEREST STATEMENT

The authors declare no potential conflicts of interest for the present paper.

## Supporting information

Supporting Information S1

## Data Availability

The data that support the findings of this study are available on request from the corresponding author. The data are not publicly available due to privacy or ethical restrictions.
