# Supplementary material for: Anaphylaxis management in a French pediatric emergency department: Lessons from the ANA‐PED study
Source: Clin Transl Allergy. 2023 Aug 1;13(8):e12289. doi: 10.1002/clt2.12289 (PMC10392053; doi:10.1002/clt2.12289)
Supplement: Supplementary file 1 — Supporting Information S1 [file CLT2-13-e12289-s001.docx]

**Electronic Repository Tables**

**Electronic Repository Table A** – International Classification of Diseases (ICD)-10 Anaphylaxis-Related Codes Used to select our included population, based on codes used at the pediatric ED of the University Hospital of Montpellier (France).

| Description | ICD-10 codes category | Extension |
| --- | --- | --- |
| Anaphylactic shock, unspecified | T78 | .2 |
| Allergic reaction, unspecified | T78 | .4 |
| Urticaria | L50 | .0 – 9 |
| Anaphylactic shock or reaction due to unspecified food | T78 | .0 |
| Quincke’s oedema | T78 | .3 |
| Upper respiratory tract hypersensitivity reaction | J39 | .3 |
| Localized oedema | R60 | .0 |
| Generalized oedema | R60 | .1 |
| Oedema, unspecified | R60 | .9 |
| Oedema of larynx | J38 | .4 |
| Allergic rhinitis | J30 | .1-9 |

**Electronic Repository Table B** – The anaphylaxis classification, base on the Ring and Messmer’s one^15^, adapted for the ICD-11 classification^16,17^.

| **Grade** | **Symptoms** |
| --- | --- |
| I | One organ system |
| II | ≥ 2 systems and *not life-threatening* |
| III | ≥ 2 systems, or *life-threatening* |
| IV | Cardiac and/or respiratory arrest |

**Electronic Repository Table C** – Univariate and multivariate logistic regression analysis by stepwise selection of factors associated with epinephrine/adrenaline prescription in children with anaphylaxis.

|  | Univariate analysis | | | Multivariate analysis | | |
| --- | --- | --- | --- | --- | --- | --- |
|  | **OR** | **95%CI** | **p-value** | **OR** | **95%CI** | **p-value** |
| Sex (F vs. M) | 1.094 | 0.61-1.97 | .7640 |  |  |  |
| Age (months) | 0.999 | 0.99-1.00 | **.**6368 |  |  |  |
| Previous clinical history with allergic disorder |  |  |  |  |  |  |
| Respiratory allergy | 1.365 | 0.68-2.76 | .3758 |  |  |  |
| Food allergy | 1.167 | 0.65-2.11 | .6091 |  |  |  |
| Drug allergy | 0.355 | 0.04-2.94 | .3372 |  |  |  |
| No previous clinical history | 0.798 | 0.44-1.44 | .4514 |  |  |  |
| Medical follow-up |  |  |  |  |  |  |
| Already followed for an allergy | 1.648 | 0.92-2.97 | **.0953** |  |  |  |
| Time and date of arrival at the emergency room |  |  |  |  |  |  |
| Weekend (Saturday-Sunday) | 1.064 | 0.56-2.03 | .8518 |  |  |  |
| Night (8pm-7.59am | 0.593 | 0.33-1.08 | **.0851** |  |  |  |
| Skin, subcutaneous tissue and mucosa |  |  |  |  |  |  |
| Localized urticaria | 6.853 | 1.29-36.30 | **.0237** | 12.673 | 1.98-81.15 | **.0074** |
| Generalized urticaria | 0.691 | 0.36-1.33 | .2660 |  |  |  |
| Localized angioedema | 1.152 | 0.57-2.34 | .6963 |  |  |  |
| Generalized angioedema | 1.499 | 0.65-3.45 | .3418 |  |  |  |
| Respiratory tract |  |  |  |  |  |  |
| Sensation of dyspnea | 0.650 | 0.35-1.20 | **.1660** |  |  |  |
| Wheezing / bronchospasm | 2.750 | 1.49-5.06 | **.0012** | 2.941 | 1.53-5.66 | **.0012** |
| Rhino-conjunctivitis | 2.152 | 1.04-4.44 | **.0380** |  |  |  |
| Gastrointestinal tract |  |  |  |  |  |  |
| Diarrhea | 0.495 | 0.11-2.33 | .3731 |  |  |  |
| Vomiting or nausea | 0.672 | 0.36-1.25 | **.2116** |  |  |  |
| Cardiovascular system |  |  |  |  |  |  |
| Hypotension | 0.610 | 0.26-1.41 | **.2484** |  |  |  |
| Central nervous system |  |  |  |  |  |  |
| Feeling of uneasiness | 0.621 | 0.26-1.51 | **.**2923 |  |  |  |
| Treatments received in ED |  |  |  |  |  |  |
| Glucocorticoids IV or IM | 1.715 | 0.82-3.59 | **.1533** |  |  |  |
| Glucocorticoids oral | 1.828 | 0.97-3.43 | **.0605** | 2.574 | 1.26-5.28 | **.0099** |
| Antihistamine IV | 1.318 | 0.53-3.25 | .5490 |  |  |  |
| Antihistamine oral | 1.443 | 0.73-2.86 | .2936 |  |  |  |
| Epinephrine/adrenaline IM | 1.875 | 0.91-3.89 | **.0907** |  |  |  |
| Epinephrine/adrenaline Inhalation | 1.944 | 0.90-4.22 | **.0931** |  |  |  |
| Oxygen | 2.112 | 0.75-5.94 | **.1565** |  |  |  |
| Salbutamol | 1.617 | 0.80-3.25 | **.1774** |  |  |  |
| Discharge care post-emergency (medical advice) | 2.600 | 1.41-4.78 | **.0021** | 2.550 | 1.34-4.85 | **.0043** |

**Electronic Repository Table D** – Univariate and multivariate logistic regression analysis by stepwise selection of factors associated with the referral to an allergist in children with anaphylaxis.

|  | Univariate analysis | | | | Multivariate analysis | | |
| --- | --- | --- | --- | --- | --- | --- | --- |
|  | **OR** | **95%CI** | **p-value** | **OR** | | **95%CI** | **p-value** |
| Sex (F vs. M) | 0.545 | 0.31-0.95 | .**0327** | 0.522 | | 0.29-0.94 | **.0309** |
| Age (months) | 0.999 | 0.99-1.00 | .5300 |  | |  |  |
| Time and date of arrival at the emergency room |  |  |  |  | |  |  |
| Weekend (Saturday-Sunday) | 1.296 | 0.69-2.43 | .4176 |  | |  |  |
| Night (8pm-7.59am | 0.993 | 0.57-1.72 | .9800 |  | |  |  |
| Skin, subcutaneous tissue and mucosa |  |  |  |  | |  |  |
| Localized urticaria | 0.704 | 0.15-3.23 | .6518 |  | |  |  |
| Generalized urticaria | 0.542 | 0.27-1.07 | **.0773** |  | |  |  |
| Localized angioedema | 1.152 | 0.57-2.34 | **.**6963 |  | |  |  |
| Generalized angioedema | 1.389 | 0.58-3.32 | .4595 |  | |  |  |
| Respiratory tract |  |  |  |  | |  |  |
| Sensation of dyspnea | 0.705 | 0.40-1.23 | **.2189** |  | |  |  |
| Wheezing / bronchospasm | 1.371 | 0.75-2.52 | .3081 |  | |  |  |
| Rhino-conjunctivitis | 1.900 | 0.85-4.25 | **.1180** |  | |  |  |
| Gastrointestinal tract |  |  |  |  | |  |  |
| Diarrhea | 0.737 | 0.23-2.40 | .6101 |  | |  |  |
| Vomiting or nausea | 1.947 | 1.07-3.54 | **.0291** |  | |  |  |
| Cardiovascular system |  |  |  |  | |  |  |
| Hypotension | 1.445 | 0.68-3.09 | .3417 |  | |  |  |
| Central nervous system |  |  |  |  | |  |  |
| Feeling of uneasiness | 1.584 | 0.70-3.59 | .2701 |  | |  |  |
| Convulsions | 0.173 | 0.02-1.69 | **.1309** |  | |  |  |
| Treatments received in ED |  |  |  |  | |  |  |
| Glucocorticoids IV or IM | 1.819 | 0.81-4.08 | **.1464** |  | |  |  |
| Glucocorticoids oral | 1.091 | 0.62-1.92 | .7613 |  | |  |  |
| Antihistamine IV | 0.721 | 0.30-1.71 | .4577 |  | |  |  |
| Antihistamine oral | 0.646 | 0.34-1.23 | **.1833** |  | |  |  |
| Epinephrine/adrenaline IM | 1.900 | 0.85-4.25 | **.1180** |  | |  |  |
| Epinephrine/adrenaline Inhalation | 1.434 | 0.69-3.27 | .3923 |  | |  |  |
| Oxygen | 2.444 | 0.68-8.85 | **.1736** |  | |  |  |
| Salbutamol | 1.182 | 0.59-2.39 | .6411 |  | |  |  |
| Discharge care post-emergency (medical advice) | 4.091 | 2.26-7.42 | **<.0001** | 4.175 | | 2.28-7.63 | **<.0001** |
